# Hansen’s disease patient in Ohio successfully treated with a monthly rifampin, minocycline, and moxifloxacin regimen: a case report

**DOI:** 10.1128/asmcr.00080-24

**Published:** 2025-06-17

**Authors:** Daniella E. Sankovic, Alyssa L. Hubal, Mandy A. Alhajj, Neil W. Anderson, Sree Cherian, Praveen Gundelly, Eric M. Ransom

**Affiliations:** 1 Department of Internal Medicine, University Hospitals Cleveland Health System, Cleveland, Ohio, USA; 2 Department of Pathology, Case Western Reserve University School of Medicine 12304, Cleveland, Ohio, USA; 3 Department of Dermatology, Louis Stokes Cleveland Veterans Affairs Medical Center, Cleveland, Ohio, USA; 4 Department of Pathology, University Hospitals Cleveland Health System, Cleveland, Ohio, USA; Pattern Bioscience, Austin, Texas, USA

**Keywords:** leprosy, Hansen's disease, *Mycobacterium leprae*, AFB, acid fast bacilli, monthly therapy, treatment, RMM, mycobacteria

## Abstract

**Background:**

Hansen’s disease, also known as leprosy, is an infection of the skin and peripheral nerves that is rarely encountered in the United States. This case describes a patient without recent international travel who achieved a successful therapeutic outcome using a new monthly multidrug treatment regimen recommended by the National Hansen’s Disease Program.

**Case Summary:**

A 78-year-old male presented with peripheral neuropathy, widespread macular erythematous rash involving face, trunk, extremities, bilateral hand edema, and oligoarticular unilateral elbow and knee bursitis. A skin biopsy revealed a lymphohistiocytic infiltrate in the presence of acid-fast bacilli (AFB) without globi. No AFB were recovered from tissue culture. Direct molecular testing from the skin biopsy detected *Mycobacterium leprae*. The patient had no recent risk factors for contracting Hansen’s disease, other than travel to Florida. The patient was treated with a monthly multidrug therapy regimen of rifampin, moxifloxacin, and minocycline (RMM) and had successful clinical reduction in disease manifestations.

**Conclusion:**

Hansen’s disease is uncommon in the United States, especially in the Midwest. However, providers should include Hansen’s disease on their differential even if recent travel was only domestic, particularly to Florida. In addition, the monthly multidrug treatment regimen RMM is an effective monthly treatment alternative, especially for patients with concerns of intolerance to first-line drugs.

## INTRODUCTION

Hansen’s disease, also known as leprosy, is an infection of the skin and peripheral nerves. The World Health Organization (WHO) has suggested erasing the term “leprosy” from medical literature, suggesting instead Hansen’s disease to reduce stigmatization and discrimination (1). Hansen’s disease commonly presents with localized skin lesions that can be raised or flat, light or pigmented, and associated with sensory loss. Long-term complications result mostly from neuropathy and systemic disease manifestations. Hansen’s disease is classified along a spectrum from tuberculoid to lepromatous forms. Patients with a high degree of cell-mediated immunity and delayed hypersensitivity present on the tuberculoid end of the spectrum, with few well-demarcated lesions. Patients with no apparent immunity to *M. leprae* present on the lepromatous end of the spectrum with numerous, poorly demarcated lesions with a high bacterial load. Important to this case, Hansen’s disease is uncommon in the United States and is most associated with travel to or immigration from an endemic country. The presented case describes a patient who had not traveled to a highly endemic country for > 55 years and likely was exposed domestically during his time volunteering outdoors in Florida.

Hansen’s disease is caused by *Mycobacterium leprae* and the proposed species *Mycobacterium lepromatosis*, which would comprise the *M. leprae* complex (2). *M. leprae* is an acid-fast, obligate intracellular bacillus that cannot be cultured in artificial media in a clinical microbiology laboratory. Diagnostic testing may include the gold-standard histopathological examination of a skin biopsy or direct nucleic acid amplification testing (NAAT) (3). As for treatment, the WHO recommends a multidrug therapy of rifampin, dapsone, and clofazimine for lepromatous leprosy. However, this regimen is not well-tolerated, and clofazimine can cause significant dermatologic side effects. The presented case describes a successful outcome using a new drug regimen with rifampin, minocycline, and moxifloxacin (RMM).

## CASE PRESENTATION

**Fig 1:**
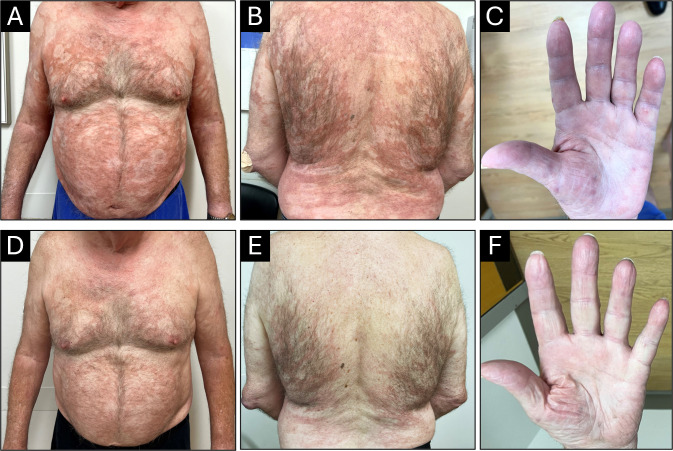
Rash and nodules as seen on front (**A**), back (**B**), and hand (**C**). The respective images post-treatment are D, E, and F.

**Fig 2:**
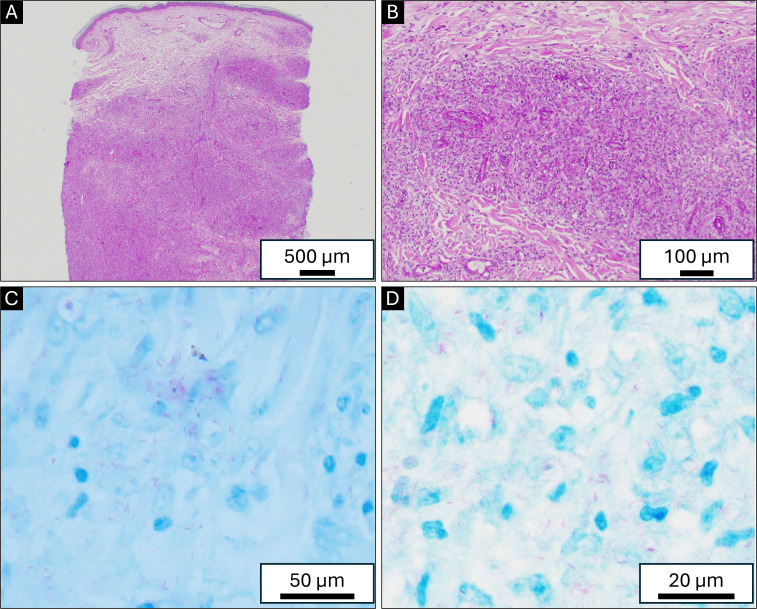
(A and B) Hematoxylin and eosin stain with interstitial lymphohistiocytic infiltrate within the superficial and deep dermis without globi. (C and D) Numerous weakly staining AFB noted among histiocytes on the acid-fast staining.

A 78-year-old male with a history of hypertension, hyperlipidemia, obstructive sleep apnea, diabetes mellitus type 2 (hemoglobin A1c at the time of diagnosis: 6.4%), and neuropathy involving both feet and legs presented to his family physician for a skin rash in February 2020. The rash was described as macular, confluent, nonpruritic, and erythematous, involving arms and legs. This was thought to be an allergic rash, and the patient was prescribed a short course of prednisone. Because the rash persisted, the patient followed up with dermatology in May 2020. The rash was then described as erythematous plaques with scaling. The patient underwent a skin biopsy that showed atrophic epidermis and superficial interstitial lymphohistiocytic infiltrate with superficial and deep perifollicular lymphohistiocytic infiltrate. These were considered non-specific findings and considered drug-related. The patient was on metformin for diabetes and valsartan for hypertension, which were both discontinued, and the rash did not improve. At this point, an infectious etiology was not considered in the differential diagnosis, and no additional workup was performed (i.e., no AFB stain on the skin biopsy specimen). An extensive workup was performed to rule out autoimmune disorders, including lupus. The patient was treated intermittently with doxycycline, triamcinolone cream, prednisone, and hydroxychloroquine with minimal response. The patient did not follow up until May 2022. Meanwhile, the rash continued to wax and wane, involving various parts of the body, including the trunk, all four extremities, and the face. In June 2022, the patient sought care with dermatology due to having nodular lesions on the thighs and legs and swelling of hands (Fig. 1A through C). The nodular lesions were biopsied, and histopathology showed an interstitial infiltrate of histiocytes with mild to moderate lymphocytic infiltrate without granuloma formation (Fig. 2). Acid-fast staining revealed numerous acid-fast bacilli (AFB) among the histiocytes. The patient had a repeat biopsy of a skin lesion for mycobacterial culture, but the AFB failed to grow after 8 weeks. The formalin-fixed paraffin-embedded (FFPE) skin biopsy specimen was sent to the molecular microbiology laboratory at the University of Washington for broad-range PCR and next-generation sequencing, which detected *M. leprae*.

The patient was treated for lepromatous leprosy with expert advice from the National Hansen’s Disease Program. At the time of diagnosis, the patient was suspected to be in lepra reaction, an inflammatory response to the AFB. The patient was started on prednisone (60 mg daily with a taper over the next 2 weeks to 10 mg) to decrease the inflammatory reaction. Following the steroid course, the patient was started on rifampin (600 mg once a month), minocycline (100 mg once a month), and moxifloxacin (400 mg once a month) for a total of 24 months. Adjunctive therapy included prednisone, methotrexate, folic acid, and vitamin D. It took several months for the rash to improve, and the patient successfully completed the 2-year course of treatment and showed improvement (Fig. 1D through F). Because there is no test of cure, additional testing was not performed. Instead, the patient was followed for the appearance of new skin lesions.

## DISCUSSION

Hansen’s disease is most commonly found in tropical and semitropical regions and is considered a neglected tropical disease (4). In 2023, the estimated number of new cases detected worldwide was 182,815 (4). According to the National Hansen’s Disease Program, only 369 new cases were reported in 2023 from the United States of America, and most (~62%) of these new cases were reported in Florida, Texas, New York, California, Arkansas, Louisiana, and Hawaii (5). Central Florida accounted for 81% of cases reported in Florida and almost one-fifth of nationally reported cases (6). Several cases in central Florida demonstrated no clear evidence of zoonotic exposure or traditionally known risk factors (7–10). Historically, new cases of leprosy were seen in patients who immigrated from leprosy-endemic areas, but there is increasing evidence of autochthonous cases of leprosy in the southern USA with no history of foreign exposure. In one study, 29 out of 39 patients did not have any history of foreign residence (8).

Zoonotic transmission of *M. leprae* has been described to occur through nine-banded armadillos (*Dasypus novemcinctus*), which are only found in the southern USA with confirmed natural infections in populations across Louisiana, Texas, Mississippi, Alabama, Florida, Georgia, and Arkansas (8–11). The prevalence of armadillo carriage varies by geographic region and method of detection, ranging from 0% to as high as 29.6% (12). Serological studies (anti-PGL I antibodies) show higher rates of exposure than histopathological methods, with prevalence rates averaging 7.1% in Louisiana and up to 17.1% in Texas. Longitudinal studies from 2005 to 2010 in Mississippi showed a stable infection prevalence of ~9.3%. In addition to zoonotic transmission, the environment may also act as a reservoir for *M. leprae,* as viable cells have been detected in soil and water from leprosy-endemic areas (12).

The two most used leprosy classifications are the Ridley-Jopling classification, which includes five types (tuberculoid type, borderline tuberculoid, borderline, borderline lepromatous, and lepromatous leprosy), and the WHO classification, which includes paucibacillary and multibacillary. The WHO classification is a clinical classification that helps in making treatment decisions without the need for skin biopsy or histopathologic examination. Patients with leprosy can develop reactions, which are immunological phenomena that occur before, during, or after treatment. There are two lepra reactions, type I or reversal reaction and type II or erythema nodosum leprosum. Type 1 reaction is recognized clinically by increased erythema, edema, and occasional ulceration of pre-existing cutaneous plaques or nodules. Type II is clinically recognized by new painful, tender, and erythematous subcutaneous nodules, which are thought to be a result of antigen-antibody complex deposition with subsequent activation of complement (13).

Of note, the patient presented here is a lifelong Ohio resident with no current known risk factors for contracting Hansen’s disease. His last known potential for international exposure was more than 55 years ago, with travel to Panama and Vietnam in the late 1960s. The patient spends 3 months a year in Volusia County in Florida, and volunteers for the forest department picking up trash on the beaches, which may be the epidemiological link. The patient had no pets and no contact with armadillos.

Early diagnosis and treatment are necessary to minimize the likelihood of irreversible nerve damage leading to permanent disability involving the hands, feet, and eyes. To diagnose, a full-thickness skin biopsy (≥4 mm punch biopsy) should be taken from the leading edge for histopathology and NAAT, because the margin of the lesion has an increased yield for AFB and it more accurately reflects the true histologic features rather than the central clear portion where the yield is low for AFB and usually only shows scarring with no inflammatory cells (14). Pathological findings can be used to distinguish tuberculoid leprosy and lepromatous leprosy; lepromatous leprosy lacks granuloma formation, has minimal lymphocytes, and has abundant AFB. For patients with lepromatous disease, NAATs from biopsies have high sensitivity and specificity (in one study, 93% and 100%, respectively) (15). Importantly, *M. leprae* is not culturable, and *M. leprae* should be considered when acid-fast bacilli are seen in tissue but not cultured (14).

Globi are amphophilic mycobacteria within histiocytes. Part of the histopathologic interest with this multibacillary leprosy case was the absence of globi on histopathology (Fig. 2), which are typically identified in multibacillary cases. It is plausible this may have been related to the incubation period in this patient since *M. leprae* can have an incubation period of about 5 years, but symptoms can take 20 years or longer to appear (16–18). The case demonstrates the importance of follow-up and confirmatory testing in the setting of atypical histopathologic findings and a clinical morphological change, even in the absence of positive culture.

The current WHO recommendation for multibacillary leprosy includes a multidrug therapy with rifampin (600 mg once a month), dapsone (50 mg once daily), and clofazimine (300 mg once a month then 50 mg daily) for 12 months (19). Treatment recommendations are evolving, in part, because approximately one in five patients with Hansen’s disease develop reactive episodes of various severity, most commonly during the first year of therapy (20). Another drawback of the WHO regimen is that clofazimine is associated with skin hyperpigmentation, which can be stigmatizing. Finally, the WHO regimen can lead to medication noncompliance since medication is taken every day.

In this case, therapeutic decision-making was guided by experts at the National Hansen’s Disease Program based on a clinical trial of 10 patients (nine with multibacillary and one with pure neural leprosy) showing successful treatment with RMM (5, 21). The RMM regimen has not been adopted by WHO, in part, due to higher costs to the patient or, in some situations, the medication-providing organization. Minocycline can be used as a substitute for dapsone or clofazimine in individuals who cannot tolerate them (22). Clarithromycin is also effective and can be used as a substitute for any of the other drugs in a multiple drug regimen (22). Ofloxacin may be used in place of clofazimine for adults (22, 23). In a clinical trial of moxifloxacin in eight multibacillary leprosy patients, moxifloxacin proved highly effective (24). Importantly, the duration with the various combination treatments is largely consistent at 2 years, but reducing the frequency to 1 day a month is promising for compliance. In the presented case, the RMM regimen was used because it is simple and easily tolerated without the clofazimine hyperpigmentation risk.

A limitation of this report is the sample size of a single patient. While readers should exercise caution applying our findings broadly, published data about the new RMM regimen are limited. In addition, this report serves as a reminder to include Hansen’s disease on a differential diagnosis even if the patient has no recent travel to an endemic region. Lastly, the images of the patient’s rash and microscopy of the skin biopsy can serve as educational materials, as these are limited in the published literature.

In conclusion, the presented case highlights a successful outcome of Hansen’s disease using the RMM monthly multidrug regimen in a patient with no recent travel history to an endemic region. The case also emphasizes the diagnostic considerations when Hansen’s disease is on the differential.
